# Gut Microbiomes of Endangered Przewalski’s Horse Populations in Short- and Long-Term Captivity: Implication for Species Reintroduction Based on the Soft-Release Strategy

**DOI:** 10.3389/fmicb.2020.00363

**Published:** 2020-03-12

**Authors:** Liping Tang, Yimeng Li, Amrita Srivathsan, Yunyun Gao, Kai Li, Defu Hu, Dong Zhang

**Affiliations:** ^1^School of Nature Conservation, Beijing Forestry University, Beijing, China; ^2^Department of Biological Sciences, National University of Singapore, Singapore, Singapore

**Keywords:** Przewalski’s horse, gut microbiome, soft-release, captivity effects, fecal samples, comparative study

## Abstract

Captivity maybe the only choice for survival of many endangered vertebrates, and understanding its broad effects is important for animal management and conservation, including breeding endangered species for subsequent release. Extreme environmental changes during captivity may influence survival ability in the wild. Captivity decreases gut bacterial diversity in a wide range of animals. However, most studies directly compare animals living in captivity with those in the wild, and there is a lack of understanding of effects of gradient shift in lifestyle during species reintroduction based on the soft-release strategy, which involves a confinement period in a field enclosure. Here, we used 16S rRNA amplicon sequencing to analyze gut microbiomes of 11 captive and 12 semi-wild Przewalski’s horses (PH; *Equus ferus przewalskii*) under the same captivity environment, using fecal samples. A subset of samples with abundant extracted DNA (including 3 captive and 3 semi-wild individuals) was selected for whole-genome shotgun sequencing. We found that community diversity did not differ between the semi-wild PH and captive PH, but the semi-wild PH had significantly higher bacterial richness than those in captivity. Relative abundances of all dominant phyla were similar across the semi-wild or captive horses, while those of the non-dominant phyla Tenericutes and Proteobacteria were significantly higher in semi-wild PH than in captive PH. Beta diversity results indicated that bacterial communities of captives and semi-wild horses were clearly separated distinct when considering only composition. Functional profiling of the microbiomes revealed that the semi-wild and captive gut microbiomes were largely similar. However, semi-wild horse microbiomes had higher abundance of bacterial genes related to core metabolic processes, such as carbohydrates, amino acids, and nucleic acid metabolism. The study revealed that semi-wild PH could retain specific non-dominant bacteria and harbor a more diverse microbiome than the captive counterpart, and thus have higher metabolic potential to utilize the complex plants efficiently. These results indicate that change in host lifestyle may play a role in microbiome differentiation in the process of reintroduction, suggesting that a short period of time in captivity is acceptable for PH from the perspective of maintaining the richness of intestinal bacterial flora to some extent.

## Introduction

The Przewalski’s horse (PH; *Equus ferus przewalskii*) is classified as an endangered species in the IUCN Red List (King et al., 2015). PHs are important in culture and biodiversity conservation as they have a unique adaptive evolutionary history, which is different from that of domestic horses (Oakenfull et al., 2000; Gaunitz et al., 2018). Captive breeding, one of the primary methods used to conserve rapidly declining species (Conde et al., 2011), played a major role in the recovery of PHs whose threat level was reduced from extinct in the wild to endangered (Conde et al., 2011). It has been projected that the global decline in biodiversity is leading to a mass extinction event (Barnosky et al., 2011). Captive breeding maybe the only short-term choice for survival of many endangered vertebrates (Conway, 2015).

However, maintaining and releasing captive populations can be challenging because captive environments are very different from those of the wild, which can impose multiple selection pressures, causing phenotypic plasticity and genetic adaptation (Schulte-Hostedde and Mastromonaco, 2015). Particularly, reintroduced animals may face a high extinction risk when released into sites with harsh and unpredictable environments. For example, the Great Gobi B Strictly Protected Area in Mongolia lost on average 60% of PHs during the severe winter of 2009/2010 (Kaczensky et al., 2011). To enhance the ability of a founder group to settle during release, soft release strategies, wherein animals experience a period of confinement at the release site, are regularly used for animal reintroduction (Clarke et al., 2002; Hardman and Moro, 2006). In Xinjiang, China, the first captive PHs were released into the Kalamaili Nature Reserve (KNR) in 2001 (Chen et al., 2008; Xia et al., 2014). This reserve is located in the northeastern part of the Junggar Basin with a harsh continental-type local climate. The dominant plant species in KNR include shrubs (*Haloxylon* spp. and *Tamarix* spp. etc.), forbs (*Anabasis* spp., *Krascheninnikovia* spp., *Artemisia* spp. Etc.), and grasses (mainly *Stipa* spp. and *Carex* spp.) (Zhang et al., 2015). Before driven into captivity, *Stipa*, *Pamirian*, *Artemisia* and *Anabasis* are the major food plant genera of Przewalski’s horses (Meng, 2007). PHs range freely from spring to late fall, and then are driven back into captivity during the severe winters to allow them to adapt to the local environment step by step (Chen et al., 2008; Xia et al., 2014).

Recent studies show that captivity can strongly affect animal microbiomes, reducing symbiotic bacterial diversity and pathogen resistance (Clayton et al., 2016; Li et al., 2017; Mckenzie et al., 2017; Lavoie et al., 2018). Trillions of microbial symbionts inhabit the mammalian gastrointestinal tract, significantly influencing many aspects of host health, including immunity, digestion, and metabolism (Sommer and Backhed, 2013). An altered gut microbiome may have negative effects on health and reproductive output in captive and released populations (Mckenzie et al., 2017; Lavoie et al., 2018). It is suggested that the differences between microbiomes in reintroduced and wild individuals is one of the potential causes for failure of species reintroduction programs (Redford et al., 2012). Therefore, clarifying the effects of captivity on gut bacterial communities has important implications for animal conservation and management. However, little is known about changes in microbial communities in endangered animals during soft-release strategy-based reintroduction processes.

Both host intrinsic factors and environmental factors are associated with the variation in gut bacterial communities (Wang et al., 2018; Knowles et al., 2019). Captive environments are extremely different from those of the wild. Notable changes include restricted diet and reduced range, habitat, cohabitation, and higher exposure to human-associated microbes (Hyde et al., 2016; Mckenzie et al., 2017). Multiple well-controlled animal and human studies have demonstrated that these environmental factors can strongly affect gut microbiome composition (Song et al., 2013; Amato et al., 2015). In particular, diet is a major contributor to gut microbial variation (Muegge et al., 2011; Li et al., 2013; Amato et al., 2015; Carmody et al., 2015). Previous studies have revealed a decreased bacterial diversity in captive PH, which is likely due to shift in diet (Li et al., 2019). A key issue, then, is whether the hosts have the ability to retain diverse bacteria when confined for a short-term.

One obstacle to a better understanding of the effects of captivity on gut microbiome variation is the numerous potential factors that could explain naturally occurring patterns. In most comparative studies, captive and wild members of a species often live in different habitats. For example, previous studies showed that gut bacteria in PH form a distinct, more diverse community than those in domestic horses (Mckenzie et al., 2017; Metcalf et al., 2017) and captive PH (Li et al., 2019), but the influence of environmental change (e.g., diet) was not excluded. Thus, these studies could not determine whether the reduced bacterial diversity in captivity is due to decreased contact with varying diets and environmental substrates (e.g., soil and aquatic systems) that can transmit transient bacteria (Kohl and Dearing, 2014; Mckenzie et al., 2017) or changes in host physiology under captivity. Contrasting results have been found when this has been examined. For example, wild-caught rodents have been shown to retain a substantial proportion of their preexisting gut bacteria after being brought back into captivity (Kohl and Dearing, 2014). In contrast, reduced diversity of intestinal bacterial community was observed in the Atlantic cod after entering captivity (Dhanasiri et al., 2010).

Given that the gut microbiome plays an important role in an animal’s health and has the potential to improve the success rate of reintroduction programs under soft-release strategy, it is important to explore the microbiomes of the wild animals undergoing short-term confined rearing. In the current study, captive and semi-wild populations of PH under the same diet were compared via fecal 16S amplicon sequencing and shotgun sequencing.

## Materials and Methods

### Sample Collection

The experiment employed 12 captive (six male and six female) and 11 semi-wild (six male and five female) PHs; fecal samples were collected from these animals during December 11–24, 2017, when they were both in captivity. Captive PHs were maintained for the full year in stalls at the Xinjiang Uygur Autonomous Region Wild Horse Breeding Research Center (WHBRC), located in Jimsar County, Changji City, Xinjiang, China (44°12′12″N, 88°44′26″E). Semi-wild PHs were kept in separate stalls only in winter from first snow (usually in November) to next March near the KNR field station, in northeastern Junggar Basin, Xinjiang (45°14′10.58N″, E89°2′42.38″). The semi-wild horses had been in the captivity more than 20 days before the samples were collected. The two sites are about 150 km apart (Xia et al., 2014). Alfalfa and water were available daily for both populations during sampling or since 20 days before sampling. Average temperature during the sample period was −14 ± 6°C. Fresh fecal samples were collected and labeled. Within 30 min of collection, samples were sent to the laboratory for DNA extraction.

This study was carried out in accordance with the guideline of the Institution of Animal Care and the Ethics Committee of Beijing Forestry University. Fecal sample collection was approved by the WHBRC and KNR.

### DNA Extraction

DNA was extracted from fresh samples using the Qiagen fast stool mini kit (QIAGEN Sciences, United States) following the standard protocol, with some modifications. Specifically, 0.25 g silicon beads (MOBIO Laboratories, Carlsbad, CA, United States) were added into sample tubes and vortexed vigorously (Scientific Industry, Vortex Genie 2) for 3 min at the highest speed.

### 16S rRNA Genes Amplicon Sequencing and Bioinformatics Analysis

The V3–V4 region of bacterial 16S RNA was amplified from all the 23 samples using 515F/806R primers (Caporaso et al., 2011). The reaction volume was 25 μl with 1 μl template DNA, 12.5 μl taq master mix, 1 μl each of 5 μM forward and reverse primers and 9.5 μl DNase-free sterile water (CoWin Biosciences, CN). Thermocycling conditions were as follows: 95°C for 30 s; 30 cycles of 95°C for 30 s, 55°C for 30 s, 72°C for 45 s; and 72°C for 10 min. All PCR products were visualized using agarose gels (1% in TAE buffer) stained with Gel green (Biotium, United States), and purified with DNA gel extraction kit (Axygen, CN). QuantiFluor^TM^-ST fluorescent quantitative system (Promega, United States) were used to determine the DNA concentration of each PCR product. Amplicons were pooled in equal amounts and sequenced on an Illumina MiSeq 250 sequencing platform (Illumina, Inc., San Diego, CA, United States) at Majorbio Bio-pharm Technology Corporation, Shanghai, China. All raw sequences of 16S rRNA gene dataset obtained during this study were submitted to the NCBI Sequence Read Archive (accession number PRJNA558670).

Sequences were pre-processed using Fastp (version 0.20.0) (Chen et al., 2018) following criteria: the reads were truncated when the average quality score was less than 20 over a 50 bp sliding window and all trimmed reads shorter than 50 bp were discarded. Next, paired reads were merged into one sequence by Flash (version 1.2.11) (Magoc and Salzberg, 2011), with a minimum overlap length of 10 bp. All sequences were grouped into operational taxonomic units (OTUs) using the clustering program UCLUST (version 1.2.22) (Edgar, 2010) against the SILVA database pre-clustered at 97% sequence identity. The resulting OTUs were classified to different levels (phylum, class, order, family, and genus) using the Ribosomal Database Program (RDP).

### Metagenomic Shotgun Sequencing and Data Processing

Shotgun metagenomic sequencing was conducted on the same DNA extracts that were used for the 16S rRNA analyses. Six samples (including three captive individuals, and three semi-wild individuals) with high DNA quality were selected. DNA from these samples was sheared into fragments using the CovarisTM S220 System (Applied Biosystems, CA, United States). One metagenomic library was constructed for each fecal sample following a standard protocol from Illumina, and 150-bp paired-end reads were obtained using Illumina NovaSeq 6000 with insert sizes of 350 bp at Novogene Bioinformatics Institute (CN). The metagenomic datasets were submitted to the Genome Sequence Archive^1^ in BIG Data Center (Nucleic Acids Res 2019), Beijing Institute of Genomics, Chinese Academy of Sciences, under project PRJCA002018 (CRA002194).

All of the raw sequences were quality-filtered by using Fastp (version 0.19.6) (Chen et al., 2018), whereby the adapters were trimmed and the reads with length shorter than 50 bp and average quality score <20 were removed. Reads that aligned to the PH genome (GenBank accession No. GCA_000696695.1) were also removed using BWA (Li and Durbin, 2009). The clean reads were assembled using Megahit (version 1.1.2) (Li et al., 2015) with contig length more than 300 bp. A non-redundant gene catalog was constructed with CD-HIT (Fu et al., 2012) using a sequence identity cut-off of 0.9, and a coverage cut-off of 0.9 for the shorter sequences. The abundance profiles of the genes in each sample were determined by blasting high quality reads to non-redundant gene catalog with 95% identity with SOAPaligner software (Li et al., 2009). Functional genes in fecal microbiomes were annotated by mapping them against the COG (Tatusov et al., 2003) and KEGG (Kyoto Encyclopedia of Genes and Genomes) (Kanehisa and Goto, 2000) using Diamond (version 0.8.35) (Buchfink et al., 2014) (blastp searches, *e*-value ≤ 1e-5).

### Statistical Analysis

Alpha and beta diversity were calculated to determine the diversity and composition of the bacterial communities in the samples. Alpha diversity indices (Ace, Chao, Simpson, and Shannon), which were calculated in QIIME (version 1.9.1) (Caporaso et al., 2010), represent bacterial community richness and diversity. Beta diversity reflects differences in bacterial communities between two populations and was calculated using weighted and unweighted UniFrac distance metric (Hamady et al., 2009). A one-way analysis of similarity (ANOSIM) was used to determine between-group differences in bacterial communities. Taxa that explained these differences were identified with linear discriminant analysis (LDA) effect size (LefSe). Discriminative functional biomarkers were established based on a size-effect threshold of 3.0 on the logarithmic LDA score. Comparative functional profiling was conducted using STAMP (version 2.0.8) (Parks et al., 2014). Student’s *t*-test was used to determine significant differences (*P*-value < 0.05) in microbial communities and the functional compositions between semi-wild and captive horse fecal samples. *P*-values were adjusted for multiple comparisons using the false discovery rate (Benjamini and Hochberg, 1995).

## Results

### Sequence Statistics

**FIGURE 1:**
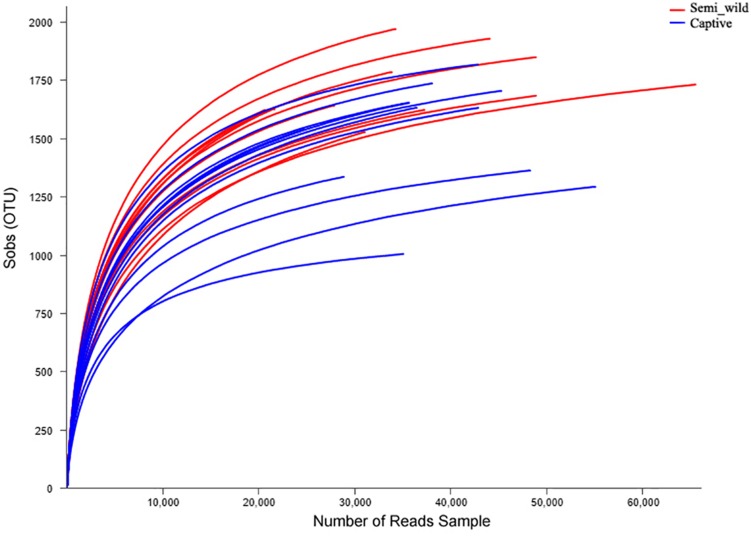
Rarefaction curves of operational taxonomic units (OTUs). The *x*-axis shows the number of reads sampled and the *y*-axis shows the observed OTUs. As the sequencing depth increases, the number of OTUs also increases. Each curve represents a sample.

We obtained 1,207,753 (52511 ± 15959) high-quality sequences corresponding to 16S from the 23 PH samples (Supplementary Table S1). Rarefaction curves revealed that the number of observed OTUs increased with sequencing depth (Figure 1). The number of OTUs detected per sample was sufficient for further analysis, as indicated by the plateauing rarefaction curves (Figure 1). The six metagenomic samples each had an average of 86,266,240 paired-end reads (Supplementary Table S2). A total of 4,962,232 genes were cataloged into a non-redundant gene catalog.

### Differences in Bacterial Community Diversity and Composition Between Semi-Wild and Captive PH

A total of 2489 OTUs were identified at 97% sequence similarity for the 16S rRNA gene datasets. These OTUs were classified into 20 phyla, 36 classes, 61 orders, 93 families, and 219 genera. Sobs (semi-wild 1724.36 ± 141.00; captive 1528.50 ± 362.41), Ace (semi-wild: 1944.89 ± 111.60; captive: 1698.76 ± 258.95) and Chao (semi-wild: 1962.57 ± 108.60; captive: 1717.82 ± 259.45) indices differed significantly between semi-wild and captive PH. Simpson (semi-wild: 0.0068 ± 0.0030; captive: 0.01 ± 0.0043) and Shannon (semi-wild: 6.07 ± 0.38; captive: 5.82 ± 0.38) indices did not differ significantly between populations (Table 1).

**TABLE 1:** Alpha diversity indexes of gut microbiome between semi-wild and captive Przewalski horse.

Alpha diversity	Semi-wild P-horse	Captive P-horse	*P*-value
Sobs	1724.36 ± 141.00	1528.50 ± 362.41	0.03
Ace	1944.89 ± 111.60	1698.76 ± 258.95	0.009
Chao	1962.57 ± 108.60	1717.82 ± 259.45	0.009
Simpson	0.0068 ± 0.0030	0.01 ± 0.0043	0.058
Shannon	6.07 ± 0.38	5.82 ± 0.38	0.129

**FIGURE 2:**
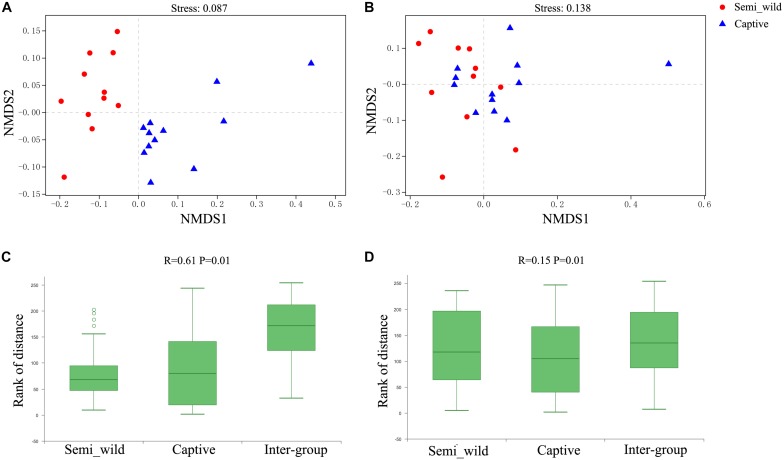
NMDS and ANOSIM Analysis. Red dots represent semi-wild Przewalski’s horses samples, and blue triangles represent captive Przewalski’s horses samples. NMDS analysis of samples representing the OTU community composition of intestinal microbiome in semi-wild and captive Przewalski’s horses. **(A)** was generated with unweighted UniFrac distance while **(B)** used weighted UniFrac distance. Stress lower than 0.2 indicates that the NMDS analysis is reliable. ANOSIM Analysis box showing the inter-group and intra-group beta distance between semi-wild and captive Przewalski’s horses. **(C)** was generated with unweighted UniFrac distance while **(D)** used weighted UniFrac distance. *R*-value range (0,1). *R*-value close to 0 represents no significant between inter-group and intra-group differences. *R*-value close to 1 shows that inter-group differences are greater than intra-group differences. *P*-value < 0.05 reflects significance of the *R* statistic.

The NMDS plot showed that samples clustered clearly by population based on the unweighted UniFrac distance metric (Figure 2A). ANOSIM results revealed significant differences in bacterial communities between semi-wild and captive PH (*R* = 0.61, *P* < 0.01; Figure 2C). However, the samples were clustered together based on weighted UniFrac method (Figure 2B), and ANOSIM results revealed only minor differences between wild and captive PH (*R* = 0.15, *P* < 0.01; Figure 2D).

**FIGURE 3:**
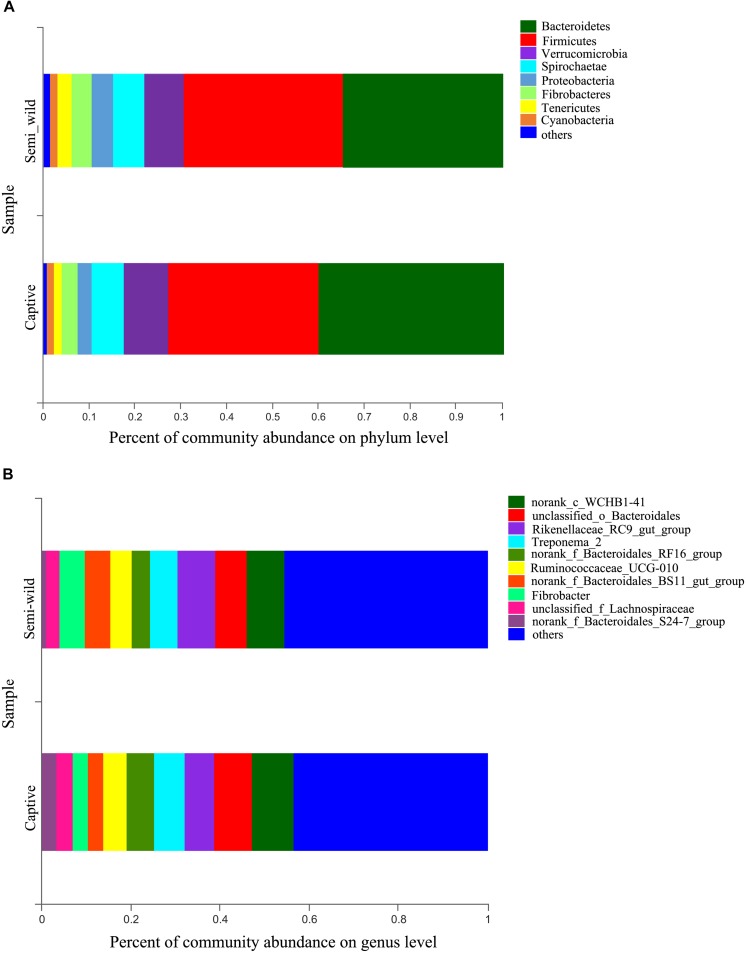
Microbial community composition. The *y*-axis represents groups and the *x*-axis represents relative abundance. **(A)** Relative abundance at phylum level. **(B)** Relative abundance at genus level; The taxa that have relative abundance less than 0.01 were combined as “others”.

**FIGURE 4:**
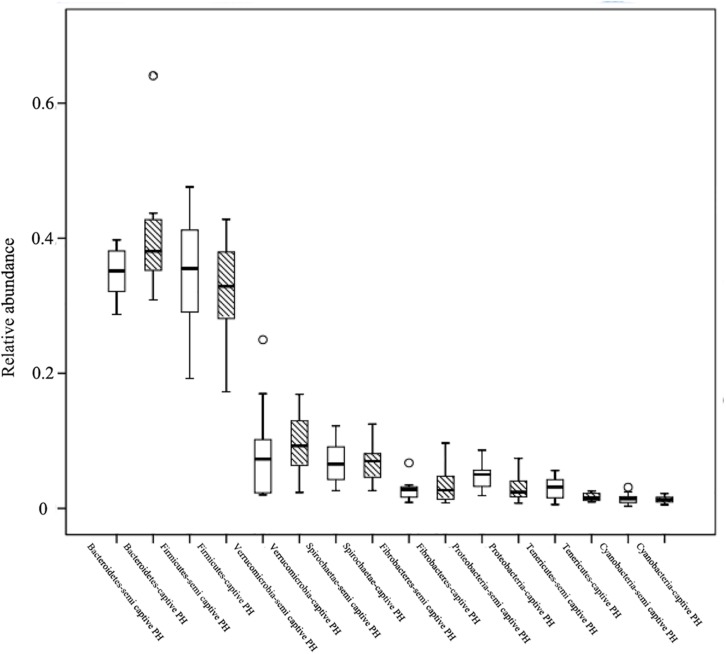
Relative abundance of bacterial phyla. Boxes represent the interquartile range (IQR) between the first and third quartiles (25th and 75th percentiles, respectively), and the horizontal line inside the box defines the median. Whiskers represent the lowest and highest values within 1.5 times the IQR from the first and third quartiles, respectively. “○” indicate outliers (greater than 1.5 times and less than three times the IQR).

At the phylum level, Bacteroidetes (semi-wild 0.35 ± 0.04, captive 0.40 ± 0.09), Firmicutes (semi-wild 0.35 ± 0.09, captive 0.33 ± 0.07), Verrucomicrobia (semi-wild 0.08 ± 0.07, captive 0.10 ± 0.04), and Spirochaetae (semi-wild 0.07 ± 0.03, captive 0.07 ± 0.03) were the dominant bacteria (>86.99% of sequences) in both semi-wild and captive PHs (Figure 3). Other phyla included Fibrobacteres (semi-wild 0.04 ± 0.06, captive 0.03 ± 0.03), Proteobacteria (semi-wild 0.05 ± 0.02, captive 0.03 ± 0.02), Tenericutes (semi-wild 0.03 ± 0.02, captive 0.02 ± 0.01), and Cyanobacteria (semi-wild 0.01 ± 0.01, captive 0.02 ± 0.01) (Figure 4). Only 0.069 and 0.12% of sequences were unclassified at the phylum level for semi-wild and captive PHs, respectively. Additionally, at genus level the microbiomes were largely similar.

**FIGURE 5:**
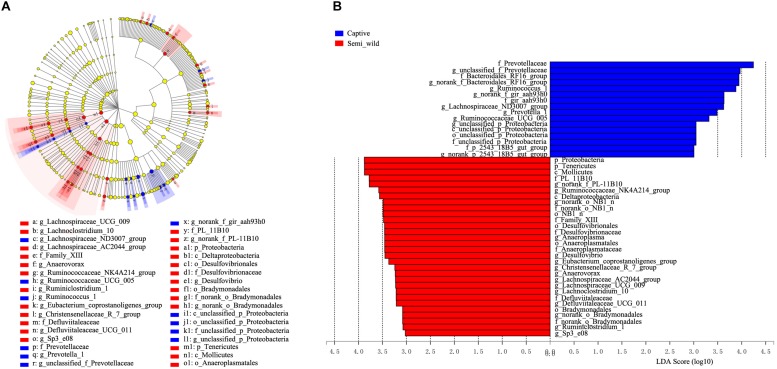
LefSe analysis. The cladogram shows the microbial taxa with significant differences between semi-wild (red) and captive Przewalski’s horses (blue). **(A)** Taxonomic hierarchies were arranged from the inside to the outside (from genus to phylum) in the cladogram. Red and blue notes represent differentially abundant taxa between groups. Yellow nodes represent taxa with no significant difference. **(B)** OTUs with significant difference that have an LDA score > the threshold value of 3. Letters in front of OTUs represent taxonomic level (p, phylum; c, class; o, order; f, family; g, genus).

**FIGURE 6:**
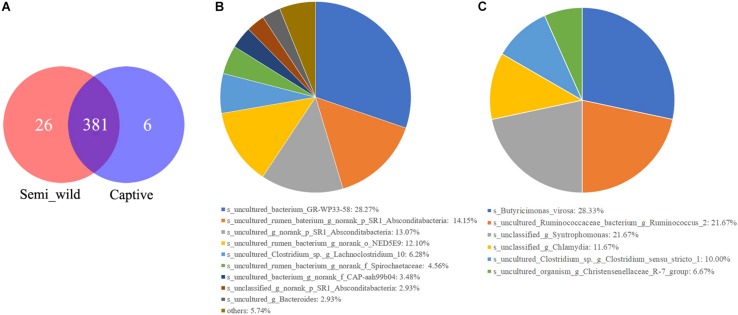
The Venn diagrams **(A)** show the numbers of species (97% sequence identity) that were shared/not shared by semi-wild and captive Przewalski’s horses. Pie charts show the numbers of species that were unique in semi-wild **(B)** and captive Przewalski’s horses **(C)**.

LEfSe analyses identified 46 bacterial taxa that explained the differences between the two groups (Figure 5A). Semi-wild PHs had significantly more Tenericutes (*P* = 0.049, LDA = 3.94) and Proteobacteria (*P* = 0.036, LDA = 3.88) than captive PHs (Figure 5B). Likewise, the semi-wild population possessed significantly more differentially abundant genera, including Ruminococcaceae_NK4A214_group (*P* = 0.002, LDA = 3.65), *Anaeroplasma* (*P* = 0.004, LDA = 3.49), and Lachnospiraceae_UCG-009 (*P* = 0.0009, LDA = 3.18). However, genera *Ruminococcus*_1 (*P* = 0.0007, LDA = 3.87), *Prevotella*_1 (*P* = 0.006, LDA = 3.55), and Ruminococcaceae_UCG_005 (*P* = 0.03, LDA = 3.36) were more abundant in captive PHs than in semi-wild PHs. Although the semi-wild and captive populations shared many species, there were 26 species unique to the semi-wild population and six species unique to the captive population (Figure 6).

### Functional Analysis of the Metagenomes

**FIGURE 7:**
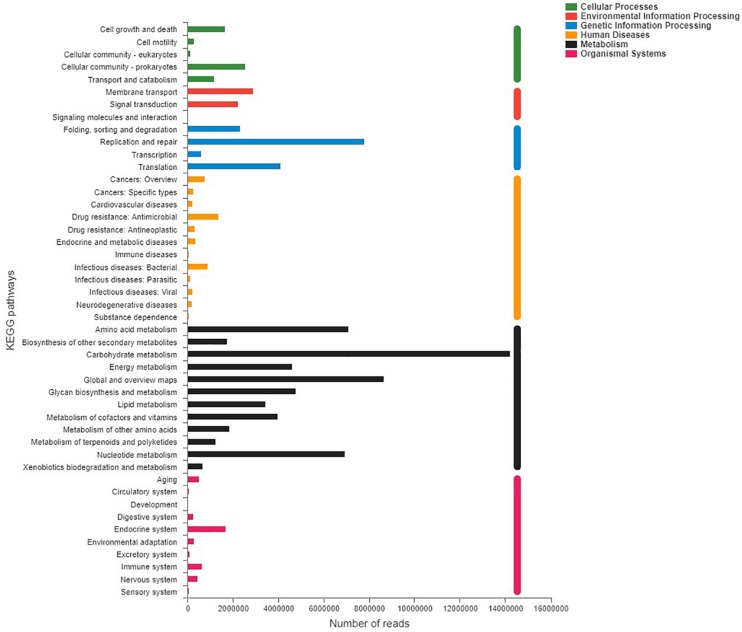
Distribution of relative abundance (normalized to the total reads for each group) for KEGG pathways (level 2) categories present in the microbial metagenome of all samples of the Przewalski’s horse. Only categories with an abundance of at least 50 are shown.

**FIGURE 8:**
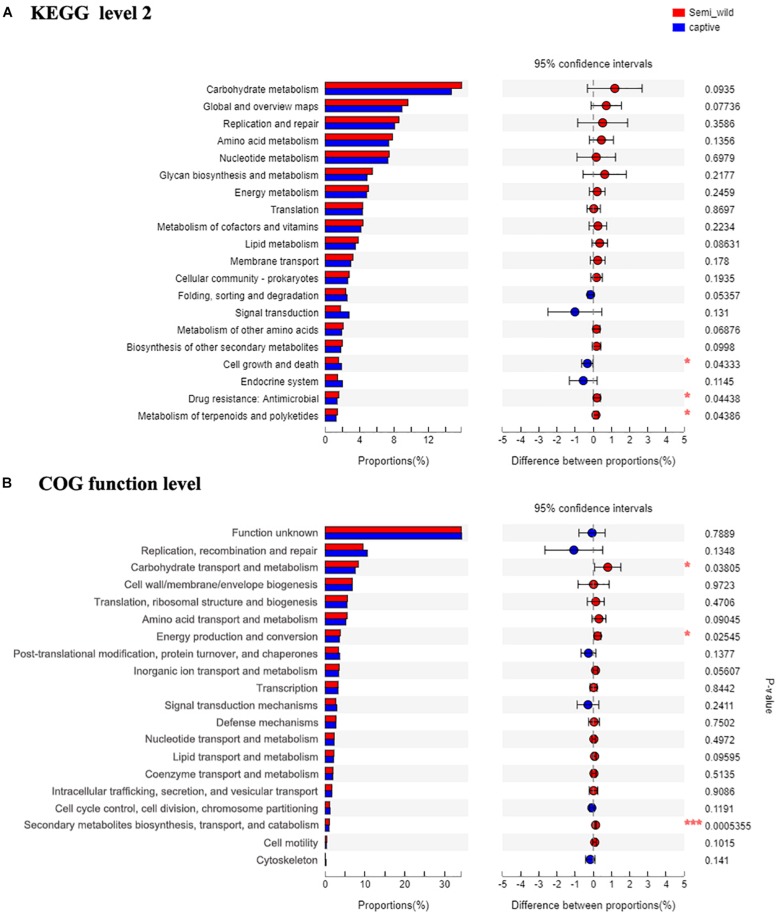
The bar plots show relative abundance (normalized to the total reads for each group) of COG categories (level function) **(A)** and KEGG pathways (level 2 function) **(B)** for the microbial metagenome of the semi-wild and captive groups. The difference between two groups is identified by STAMP software using Student’s *t*-test. *p < 0.05, **p < 0.01 and ***p < 0.001.

Shotgun metagenomic data were functionally characterized and the abundances of genes related to various KEGG pathways are shown in Figure 7. Statistical tests were conducted to investigate the differences in the abundances of genes related to various COG (level 2) and KEGG (level 2) categories (Figure 8) between the captive and semi-wild horses. This showed only few categories displayed significant differences (*P*-value < 0.05) but small fold change (<1.2X) in abundance between the two groups. Significantly higher proportions of genes affiliated to “Carbohydrate transport and metabolism,” “Energy production and conversion,” “Secondary metabolites biosynthesis, transport and catabolism,” “Drug resistance: Antimicrobial,” and “Metabolism of terpenoids and polyketides” were found in semi-wild PH. In contrast, only genes related to “Cell growth and death” was significantly enriched in the captive PH.

Although few categories showed significant difference, in general, there was a higher abundance of genes related to core metabolic functions (e.g., carbohydrate, amino acid, nucleotide, and lipid metabolism) and those related to genetic information processing (e.g., RNA translation, DNA replication and repair) in semi-wild PH. On the other hand, the genes related to pathways such as “Folding, sorting and degradation,” “Signal transduction,” “Endocrine system” were detected at slightly lower proportions in the semi-wild group compared to the captive group (Figure 8).

## Discussion

Here, we successfully characterized and compared the microbiomes of two PH populations to determine whether captivity alters microbiomes. We observed that semi-wild PHs had significantly higher bacterial OTU richness than captive PHs. However, community diversity (based on Shannon/Simpson indices) did not exhibit significant between-group variation. Differences in bacterial OTU richness between the captive and wild animals have also been observed in rodents and canids (Kohl and Dearing, 2014; Mckenzie et al., 2017). Here, we consistently observed lower richness in gut microbial community of the captive population than that of the semi-wild population, despite under the same captive environment. This result indicates that long-term captivity might has a lasting effect on PH gut microbial communities.

We observed more OTUs than reported by the previous study in the same area (Li et al., 2019), which may be the result of the different DNA extraction method. Unlike the previous study, we extracted DNA from the fresh sample directly and hence have likely avoided the potential bias caused by storage. The UPGMA clustering analysis based on the unweighted UniFrac method, which considers species presence/absent, showed a significant between-group variation in the gut microbiome composition (Figure 2A). However, when the analysis was based on a weighted UniFrac method, which considers both microbial composition and abundance, samples from the two groups clustered together (Figure 2B). This result indicates that the abundance of dominant microbiome is largely the same between captive PHs and their semi-release counterparts; this could obscure the differences in bacterial composition in terms of rare species. It is well established that dietary factors are amongst the most important factors affecting gut microbial composition (Shanks et al., 2011; Moschen et al., 2012; Bowyer et al., 2018). The difference in composition (based on unweighted UniFrac) is likely due to dietary variation that occurs when PHs are allowed to feed in the wild, indicating that preexisting bacteria in wild PHs may have been retained even after changes in diet.

The predominant bacterial phyla in both populations were Bacteroidetes and Firmicutes. The former degrades carbohydrates and other substances of high molecular weight in intestinal secretions (Thoetkiattikul et al., 2013), and the latter degrades cellulose into short chain fatty acids (Pryde et al., 2002). Our findings corroborated those of studies on domestic horses (Dougal et al., 2014) and other herbivores, such as musk deer (Li et al., 2017) and camel (Samsudin et al., 2011). Significant shifts in bacteria belonging to the phyla Bacteroidetes and/or Firmicutes along the wild to captive axis are widely recognized in mammalian hosts (Kohl and Dearing, 2014; Mckenzie et al., 2017; Metcalf et al., 2017; Pan et al., 2017). The current study showed no significant change in Bacteroidetes and Firmicutes, indicating that the short-term captivity compensated for the difference in these two predominant phyla between the captive and semi-wild states. However, among the non-dominant phyla, Tenericutes (Mollicutes) abundance was significantly higher in semi-wild than in captive PHs, similar to the observation in other species (Guan et al., 2017; Mckenzie et al., 2017). Mollicutes metabolizes sugars as an energy source (Halbedel et al., 2007), but many members cause diseases in their hosts (Christiansen and Birkelund, 1998).

At the genus level, the abundances of *Ruminococcus*_1 and Ruminococcaceae_UCG_005 were higher in captive PHs, which may be associated with fiber digestion. Higher abundance of *Ruminococcus* are associated with increased dietary hydrolyzable carbohydrates than with the grass-based diet of the domestic horse (Daly et al., 2011). Ruminococcaceae_UCG-005 was also dominant and had higher abundance in captive sika deer (Guan et al., 2017) and golden takin (Chen et al., 2017). We also observed a significant increase in the relative abundance of *Prevotella*_1 in domestic horses, which may be associated with high-grain diet and contribute to fiber, carbohydrate, simple sugar, or tannin degradation (Wu et al., 2011; Li et al., 2013) in captive horses (Fernando et al., 2010). *Prevotella* abundance was similarly elevated in captive monkeys (Hale et al., 2018), but other mammal studies reported conflicting results (Mckenzie et al., 2017). Given the role of diet in shaping the gut microbiome, especially those related to microbial fermentation in horses, the variation in abundance of bacteria under the same diet indicate that the composition of the microbiomes is not influenced by diet passively. Another explanation is that food may have a lasting or delayed effect on the microbiome.

It is not surprising that captivity with artificial forage will change the composition and function of the gut microbiome of the wild animals. The imputed relative abundances of COG and KEGG pathways revealed similarities in functional profiles of captive and semi-wild individuals. However, when compared with captive horses, semi-wild horses microbiome generally harbored slightly higher abundance of genes related to metabolic processes, such as carbohydrates, amino acids, nucleic acid, terpenoids, and polyketides metabolism. These may contribute to adaptive advantages for semi-wild horses, that allow them to utilize the diverse food efficiently even after being driven into captivity. Note, however, that these differences are minor.

Although the overall gut microbiome diversity and structure remained stable between captive and semi-wild PHs, potential zoonotic pathobionts such as *Butyricimonas virosa* and *Chlamydia* were only observed in captive horses (Figure 6). This outcome suggests that human interactions or reduction of gut microbiome diversity may contribute to pathogen infection in PHs. Because captive populations are periodically released into the KNR, we cannot ignore the risk of zoonotic pathogens spreading to wild animals. In this study, 46.74% of the sequences of 16s rRNA data were not classified to any known genera, indicating that there are still many unknown bacterial species that need to be identified, which requires further study. Although this study discusses the differences in the microbiomes between two groups of horses that differ in their captivity patterns and provides evidence that short-term captivity is applicable for PH in terms of maintaining microbiome richness to some extent, the mechanisms or processes underlying these observations are unclear, and thus, cross-sectional studies involving longitudinal sampling should be applied in the future.

Overall, the results of our study have demonstrated the effects of short-term captivity in shaping the gut microbiome variation in PH. Although no significant difference in microbiome diversity (based on Shannon/Simpson indices) were recognized between the captive and semi-wild state, PH driven from the wild tend to have a relatively higher bacterial OTU richness and harbor bacteria with greater metabolic functional potential, indicating that environment factors were not the only factors that influenced the bacterial community. Further, although our study is limited to the gut microbiome of horses, it is possible that the soft-release strategy may be acceptable for endangered species from the perspective of maintaining the richness of intestinal bacterial flora. The next important steps in this line of research include long-term individual tracing of PH in the process of release and its potential as a regular surveillance tool acceptable for endangered species reintroduction program.

## Data Availability Statement

The 16S rRNA gene datasets generated for this study can be found in the NCBI Sequence Read Archive (accession number PRJNA558670). The metagenomic datasets can be found in the Genome Sequence Archive in BIG Data Center, Beijing Institute of Genomics, Chinese Academy of Sciences, under project PRJCA002018 (accession number CRA002194).

## Ethics Statement

The animal study was reviewed and approved by the Institution of Animal Care and the Ethics Committee of Beijing Forestry University.

## Author Contributions

DZ, DH, and KL conceived of the study and participated in its design. LT, YG, and KL performed the samples collection and DNA extraction. LT and YL analyzed the data and wrote the manuscript. AS advised on the metagenomic data analysis and interpretation. AS, DZ, and DH revised the manuscript. All authors read and approved the final manuscript.

## Conflict of Interest

The authors declare that the research was conducted in the absence of any commercial or financial relationships that could be construed as a potential conflict of interest.

## Footnotes

1 http://gsa.big.ac.cn

## Supplementary Material

TABLE S1 Sequencing data corresponding to 16S from 23 fecal samples.

TABLE S2 Sequencing data of six metagenomic samples.
